# Long noncoding RNA maternally expressed gene 3 improves trophoblast dysfunction and inflammation in preeclampsia through the Wnt/*β*-Catenin/nod-like receptor pyrin domain-containing 3 axis

**DOI:** 10.3389/fmolb.2022.1022450

**Published:** 2022-10-14

**Authors:** Yue Liang, Ping Wang, Yueyang Shi, Bihong Cui, Jinlai Meng

**Affiliations:** ^1^ Shandong Provincial Hospital, Shandong University, Jinan, Shandong, China; ^2^ Department of Obstetrics and Gynecology, Shandong Provincial Hospital Affiliated to Shandong First Medical University, Jinan, Shandong, China; ^3^ Key Laboratory of Birth Regulation and Control Technology of National Health and Family Planning Commission of China, Maternal and Child Health Care Hospital of Shandong Province, Jinan, Shandong, China

**Keywords:** preeclampsia, lncRNA *MEG3*, Wnt/*β*-Catenin, NLRP3, placenta, trophoblast, inflammation

## Abstract

Inadequate trophoblastic infiltration and resulting placental hypoxia and inflammation comprise the core pathological basis of preeclampsia (PE). Maternally expressed gene 3 (*MEG3*) is known to be involved in the pathogenesis of preeclampsia by inhibiting the migration and invasion of trophoblasts and promoting their apoptosis. Nevertheless, the specific underlying downstream molecular mechanism of *MEG3* is less well characterized. In this study, we detected lower expression levels of *MEG3* and *β*-Catenin and higher expression of nod-like receptor pyrin domain-containing 3 (NLRP3) in placental tissues of pregnant women with severe preeclampsia (sPE) than in normal pregnancies. Elevated serum levels of IL-1β and TNF-α were also observed in the sPE group. Then, we established a hypoxia/reoxygenation (H/R) model to mimic preeclampsia. Similar results with sPE group were found in the H/R group compared with the control group. In addition, suppressive trophoblast proliferation, migration and invasion and increases in the apoptotic rate and inflammation were also detected in the H/R group. Notably, overexpressing *MEG3* markedly improved trophoblast dysfunction and inflammation caused by H/R. However, the effects of *MEG3* on trophoblasts, whether upregulated or downregulated, can be reversed by DKK-1 (Wnt/*β*-Catenin inhibitor) and MCC950 (NLRP3 inhibitor). The current study revealed that *MEG3* regulates trophoblast function and inflammation through the Wnt/*β*-Catenin/NLRP3 axis and provided new insights into the pathogenesis of preeclampsia.

## Introduction

Preeclampsia (PE) is a common and serious obstetrical disease that occurs in approximately 2%–8% of all pregnancies (Ives et al., 2020). It not only presents itself as a multisystem hypertensive syndrome characterized by emerging hypertension and proteinuria after the 20th week of pregnancy but also increases the risks of future cardiovascular diseases (Bokslag et al., 2016; Rana et al., 2019). Severe forms of preeclampsia are usually associated with multiorgan dysfunction and adverse pregnancy outcomes, seriously endangering the health of the mother and the fetus (Brichant et al., 2010). However, the etiology of PE is still unclear, limiting therapeutic interventions (Pennington et al., 2012). Currently, the only ways to improve outcomes for preeclampsia are prevention, timely diagnosis and placental delivery (Chappell et al., 2021). Disturbances in trophoblast migration and invasion are often regarded as the initiating event of preeclampsia (Lyall et al., 2013; Fisher, 2015). The resulting reduced placental perfusion can elicit placental oxidative stress and release a series of inflammatory cytokines into the maternal circulation (Burton et al., 2019). Eventually, a systemic inflammatory response is triggered, which directly induces the symptoms of preeclampsia.

Long noncoding RNAs (lncRNAs) are commonly defined as RNA transcripts with lengths of more than 200 nucleotides that cannot encode corresponding proteins (Robinson et al., 2020). In recent years, attention has been given to lncRNAs because their dysregulation is increasingly linked to many human diseases and is governing a variety of biological processes, such as proliferation, apoptosis, and cell migration (Ponting et al., 2009; Hombach and Kretz, 2016). The lncRNA maternally expressed gene 3 (*MEG3*), located on human chromosome 14q32, is a pregnancy-related gene (Miyoshi et al., 2000; Zhou et al., 2010). It has been proposed previously that the expression of *MEG3* was reduced in the placentas of preeclampsia patients at the trophoblast cell level. The low level of *MEG3* might be associated with failure in uterine spiral artery remodeling in preeclampsia by suppressing trophoblast cell migration, invasion and promoting apoptosis (Zhang et al., 2015; Yu et al., 2018; Wang and Zou, 2020). These results suggest that *MEG3* plays vital roles in PE. However, the specific underlying downstream molecular mechanism by which *MEG3* regulates trophoblast cells is less well characterized.

Wnt/*β*-Catenin is a highly conserved signaling pathway centered on *β*-Catenin, which is broadly implicated in numerous biological processes and human diseases (Sonderegger et al., 2010; Clevers and Nusse, 2012; Liu et al., 2022). In a variety of tumors, the Wnt/*β*-Catenin signaling pathway has been demonstrated to be activated and promotes cancer cell proliferation, growth, and survival (Damsky et al., 2011; Pramanik et al., 2015; He and Tang, 2020; Mukherjee and Panda, 2020). Moreover, Wnt/*β*-Catenin signaling has been recognized as a crucial signaling pathway in trophoblast differentiation and embryonic development (Pollheimer et al., 2006; Sidrat et al., 2021). Defects in this key signaling pathway can induce abnormalities in trophoblast invasion and subsequently lead to PE (Zhang et al., 2017; Wang et al., 2018). The nod-like receptor pyrin domain-containing 3 (NLRP3) inflammasome is an oligomeric complex that has been confirmed to be closely linked to many inflammatory and autoimmune diseases, such as atherosclerosis, gout, and diabetes mellitus (Masters et al., 2010; Davis et al., 2011; Karasawa and Takahashi, 2017; So and Martinon, 2017). NLRP3 is one of the major members of the NLRP3 inflammasome, whose activation leads to enhanced NLRP3 inflammasome activity and excessive IL-1β secretion (Shirasuna et al., 2020). Several studies have suggested that the expression of NLRP3 is substantially increased in placentas from PE and is tightly related to the well-established pathogenesis of preeclampsia, such as sterile inflammation and oxidative stress (Weel et al., 2017; Nunes et al., 2021). These data indicated a role for placental NLRP3 involvement in PE, but the detailed mechanism remains incompletely understood.

In this study, we first measured the expression pattern of the lncRNA *MEG3*, *β*-Catenin, and NLRP3 in placental tissues. To validate the results of the PE placenta, we leveraged hypoxia/reoxygenation (H/R)-challenged human trophoblasts as an *in vitro* model to show that upregulation of *MEG3* contributed to enhanced trophoblast proliferation, migration and invasion and attenuated the apoptotic rate and inflammatory status through the Wnt/*β*-Catenin/NLRP3 axis.

## Materials and methods

### Patients and sample collection

Twenty patients with severe preeclampsia (sPE) and 20 normotensive pregnant women who delivered by cesarean section at Shandong Provincial Hospital between April 2019 and September 2021 were enrolled in the current study. PE was defined as the presence of blood pressure ≥140/90 mmHg after 20 weeks of gestation and urinary protein level ≥300 mg/24 h. According to the criteria of NHBPEP (2000), diagnosis of sPE was considered when the following criteria were present: blood pressure ≥160/110 mmHg, proteinuria ≥2.0 g/24 h or + 2 dipstick, serum creatinine >1.2 mg/dl, acute pulmonary edema, HELLP syndrome, or eclampsia. Exclusion criteria included maternal history of hypertension, premature rupture of membranes, chemical dependency, diabetes mellitus, renal diseases, autoimmune and other complications that may lead to hypoxic changes.

Placental tissues were taken from the center area around the umbilical cord immediately after cesarean section, avoiding regions of infarct and calcification. The specimens were then washed in chilled phosphate-buffered saline (PBS) and stored at −80°C until further use. Maternal venous blood was collected prior to delivery, followed by serum separation and storage at −80°C.

The clinical data of all pregnant women were retrospectively collected from medical records. This study was conducted in accordance with the Declaration of Helsinki and was approved by the Ethics Committee of the Shandong Provincial Hospital (2020-805). All participants signed written informed consent forms. The clinical features of all patients in the sPE and normal groups are shown in Table 1.

**TABLE 1:** Clinical features of patients in the normal and sPE groups.

Category	Normal (*n* = 20)	sPE (*n* = 20)	*p* value
Maternal age (years)	28.95 ± 0.87	29.9 ± 1.04	NS
Gestational age at birth (weeks)	37.72 ± 0.63	35.56 ± 0.50	*
Gravidity	1.40 ± 0.11	1.50 ± 0.14	NS
BMI (kg/m^2^)	26.02 ± 0.81	28.87 ± 0.55	**
SBP (mmHg)	111.40 ± 2.52	173.60 ± 3.47	****
DBP (mmHg)	78.59 ± 1.35	113.90 ± 2.14	****
Proteinuria (g/24 h)	0.04 ± 0.01	3.24 ± 0.19	****
Neonatal birth weight (g)	3,397 ± 118.5	2,721 ± 127.9	***

BMI, body mass index; SBP, systolic blood pressure; DBP, diastolic blood pressure. Each value is represented as the mean ± SEM. NS, non significant; *p < 0.05; **p < 0.01; ***p < 0.001; ****p < 0.0001.

### Cell culture and treatment

HTR8/SVneo cells (Shanghai Zhongqiaoxinzhou Biological Technology Co., Ltd., Shanghai, China) were cultured in RPMI 1640 (Gibco) supplemented with 10% fetal bovine serum (FBS, Gibco) at 37°C in an atmosphere of 20% O_2_ and 5% CO_2_. To construct an *in vitro* hypoxia/reoxygenation model, cells were maintained in 2% O_2_, 93% N_2_, and 5% CO_2_ for 8 h (hypoxia) before being returned to standard culture conditions (20% O_2_; reoxygenation) for 16 h (Hung et al., 2010). Recombinant human Dickkopf-1 (DKK-1; 100 ng/ml) and MCC950 (1 μM) were purchased from PeproTech (NJ, United States) and MedChemExpress (NJ, United States), respectively.

### Plasmid construction and transfection

pcDNA-*MEG3*, sh-*MEG3* and their corresponding negative controls, which were referred to as pcDNA-NC and sh-NC, were designed and synthesized by Shanghai GeneChem Medical Technology Co., Ltd. (Shanghai, China). When the cell confluence reached approximately 50%, the abovementioned sequences were transiently transfected into HTR8/SVneo cells using Lipofectamine 3000 (Invitrogen, CA, United States) in Opti-MEM culture medium (Gibco) according to the manufacturer’s instructions. After 6 h, the transfection was replaced with 2 ml RPMI 1640 medium containing 10% FBS, and the cells were further cultured for 48 h and for subsequent experimentation.

### Quantitative real-time PCR

Total RNA was extracted from the placental tissues or cells using TRIzol reagent (Accurate Biotechnology, Hunan, China), and a Nanodrop 2000 spectrophotometer (NanoDrop, Thermo Fisher Scientific, MA, United States) was used to measure the purity and concentration of the extracted RNA. One microgram of RNA was reverse transcribed into complementary DNA (cDNA) using a reverse transcription kit with gDNA removal reagent (Accurate Biotechnology, Hunan, China). Subsequently, we conducted quantitative real-time PCR (qRT‒PCR) to detect the relative expression level of the target gene using a SYBR^®^ Green Pro Taq HS Premix qPCR kit (Accurate Biotechnology, Hunan, China) on a Light Cycler 480 (Roche, Switzerland). Glyceraldehyde-3-phosphate dehydrogenase (GAPDH) was considered to be an endogenous control, and the original data were calculated by the 2-ΔΔCt method. The primer sequences used in this study are listed in Table 2.

**TABLE 2:** Primer sequences for qRT‒PCR.

Gene	Primer sequence (5′-3′)
MEG3	Forward: TGC​TGC​CCA​TCT​ACA​CCT​CAC
	Reverse: GTC​CTC​TTC​ATC​CTT​TGC​CAT​CCT
β-Catenin	Forward: GGA​CCA​CAA​GCA​GAG​TGC​TGA
	Reverse: TTC​TGA​ACA​AGA​CGT​TGA​CTT​GGA
NLRP3	Forward: GAT​CTT​CGC​TGC​GAT​CAA​CA
	Reverse: GGG​ATT​CGA​AAC​ACG​TGC​ATT​A
GAPDH	Forward: GCA​CCG​TCA​AGG​CTG​AGA​AC
	Reverse: TGG​TGA​AGA​CGC​CAG​TGG​A

### Western blot analysis

Total protein in placental tissues and cells was extracted with RIPA buffer containing protease inhibitors (Thermo Fisher Scientific, MA, United States) and quantified by a BCA protein assay kit (Solarbio, Beijing, China). Equal amounts of protein were separated by 10% SDS‒PAGE (Shanghai Epizyme Biomedical Technology Co., Ltd., Shanghai, China) and transferred onto polyvinylidene fluoride (PVDF) membranes. After blocking with 5% skim milk powder at room temperature for 1 h, the membranes were incubated with the following primary antibodies (all from Abcam, Cambridge, MA, United States): rabbit monoclonal antibodies against *β*-Catenin (ab32572, 1:5000), NLRP3 (ab263899, 1:1000), c-Myc (ab32072, 1:1000), Vimentin (ab92547, 1:1000), Bax (ab32503, 1:1000), Bcl-2 (ab32124, 1:1000), and GAPDH (ab181602, 1:5000) overnight at 4°C on a shaker. Then, HRP-conjugated AffiniPure goat anti-rabbit IgG (1:5000, Proteintech, Wuhan, China) diluted in 5% skim milk powder was added for 1 h of incubation at room temperature. Finally, the transferred membrane was covered with Immobilon Western Chemiluminescent HRP Substrate (Millipore, Billerica, MA, United States), and images were developed using an Amersham Imager 680 (GE, United States). ImageJ software (NIH) was employed for quantitative analysis of protein bands. GAPDH served as the internal reference.

### Immunofluorescence

For placental tissues, paraffin-embedded sections were subjected to a standard deparaffinization and rehydration process and incubated in citric acid for antigen retrieval. After that, the sections were blocked with 1% bovine serum albumin (BSA) at 37°C for 30 min and then incubated with primary NLRP3 antibody (1:100, Proteintech, Wuhan, China) or *β*-Catenin antibody (1:500, ab32572, Abcam) at 4°C overnight. As a secondary antibody, fluorescein isothiocyanate-conjugated goat anti-rabbit antibody (1:500, Proteintech, Wuhan, China) was added for incubation at room temperature for 50 min. Nuclei were stained with DAPI (Solarbio, Beijing, China) under the conditions of protection from light for 10 min, and then the sections were sealed with antifading mounting medium (Solarbio, Beijing, China). Immunofluorescent images were visualized with confocal fluorescence microscopy (Leica, Germany), and the pixel intensity of the immunoreactive signal was measured using ImageJ (NIH).

### Enzyme-linked immunosorbent assay

Serum samples and culture media were centrifuged for supernatant collection and stored in a −80°C freezer for subsequent use. Human IL-1β and TNF-α enzyme-linked immunosorbent assay (ELISA) kits (Hangzhou Multi Sciences Biotechnology Co., LTD., Hangzhou, China) were employed to detect the levels of the cytokines IL-1β and TNF-α following the manufacturer’s instructions. The optical density (OD) value of each well was measured, and the concentration of cytokines was calculated by the standard curve generated from the standard solutions.

### Wound healing assay

The migratory ability of HTR8/SVneo cells was determined using the scratch-wound healing method. In short, cells were seeded in six-well culture plates and cultured for 24 h to ensure that the cells adhered to the wall. A wound-like gap was made on the cell layers using a 200-μl sterile pipette tip and rinsed three times with PBS. Then, the cells were cultured in serum-free RPMI-1640 medium to reduce the effect of cell proliferation on migration. Randomly selected fields were photographed under an inverted microscope (Zeiss, Oberkochen, Germany) at 0 and 48 h. ImageJ software was used to measure the scratch rate.

### Transwell assay

A transwell chamber (Corning, NY, United States) with a membrane pore diameter of 8 µm was used to conduct the transwell assay. The membrane was covered with 50 µl Matrigel (BD Biosciences, NJ, United States) diluted with serum-free RPMI 1640 medium at a ratio of 1:8. A cell suspension of 1 × 10^5^ cells in 200 µl serum-free RPMI-1640 medium was plated onto the upper chamber, and 600 µl complete medium containing 10% FBS was added to the lower chamber. After 48 h of incubation, the noninvading cells on the upper surface of the membrane were removed, while the invading cells were fixed in methanol, stained with 0.1% crystal violet, and counted microscopically.

### CCK-8 proliferation assay

HTR8/SVneo cells grown to the log phase were seeded in 96-well plates at a density of approximately 1 × 10^3^ cells per well and were cultured for 0, 24, 48, and 96 h. Then, 10 µl of CCK-8 reagent (MedChemExpress, NJ, United States) was added to each well, and the incubation was continued in the incubator at 37°C for 2 h. The absorbance value at 450 nm of all samples was determined using a microplate reader (Thermo Fisher Scientific, MA, United States) and then used to plot the growth curve of cells.

### Flow cytometric analysis of apoptosis

Cell apoptosis analysis was performed using the Annexin V-fluorescein isothiocyanate (FITC) Apoptosis Detection Kit (BD Biosciences, NJ, United States). Cells were detached from the culture plates with trypsin without EDTA (Gibco) and washed twice in PBS. Next, 100 µl binding buffer was added to resuspend the cells, followed by 5 µl FITC Annexin V and 5 µl propidium iodide (PI) solution for a 20 min incubation in the dark. Finally, the cell suspension was mixed with 400 µl binding buffer and examined using a BD FACSCalibur (BD Biosciences, NJ, United States). The data were analyzed by FlowJo software (Tree Star, San Carlos, CA, United States).

### Statistical analysis

All data are presented as the mean ± SEM and were analyzed by using GraphPad Prism 7 software (GraphPad Software Inc., San Diego, CA, United States). The data conforming to normal distribution and homogeneity of variance between two groups were analyzed by using an unpaired *t* test. Comparisons among multiple groups were performed employing one-way analysis of variance (ANOVA), followed by Tukey’s multiple comparison *post hoc* analysis. A *p* value of less than 0.05 was considered an indicator of statistical significance.

## Results

### Maternally expressed gene 3 and *β*-Catenin expression levels are low and nod-like receptor pyrin domain-containing 3 expression is high in the placental tissues of severe preeclampsia patients

**FIGURE 1:**
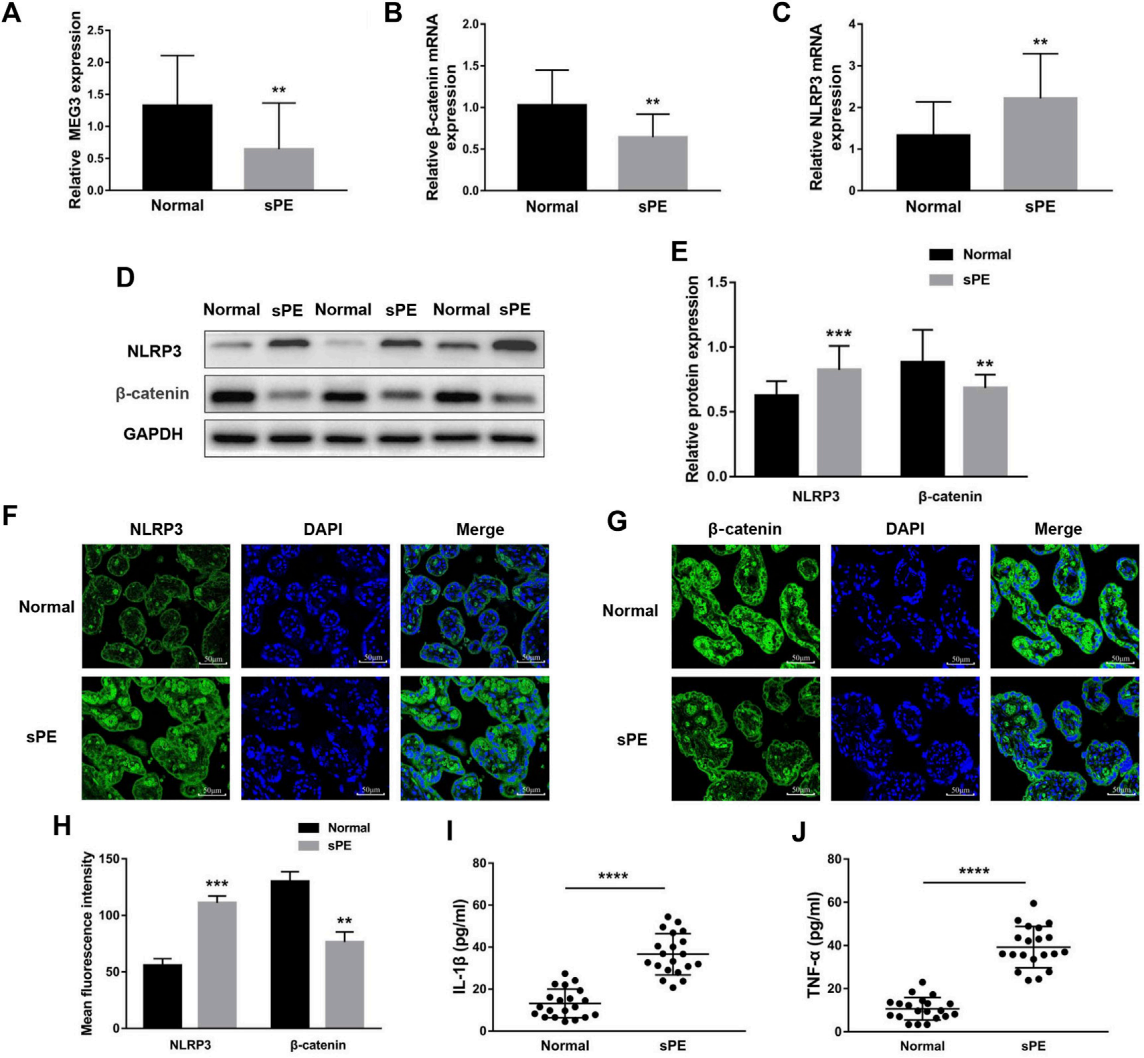
Expression of *MEG3*, *β*-Catenin and NLRP3 in the placentas of normal pregnancies and sPE patients. The relative mRNA levels of *MEG3* **(A)**, *β*-Catenin **(B)** and NLRP3 **(C)** were assessed by qRT‒PCR. **(D,E)** The relative protein levels of *β*-Catenin and NLRP3 were detected by Western blot. GAPDH was used as an endogenous control. The expression levels of NLRP3 **(F)** and *β*-Catenin **(G)** in placental tissues were evaluated by immunofluorescence (IF). **(H)** Semi-quantitative analysis of mean fluorescence intensity. The serum levels of IL-1β **(I)** and TNF-α **(J)** were detected by ELISA. *n* = 20/group. **p < 0.01; ***p < 0.001; ****p < 0.0001.

To establish the relationships between *MEG3*, *β*-Catenin, NLRP3 and the pathogenesis of preeclampsia, we first measured the expression levels of *MEG3*, *β*-Catenin and NLRP3 in placental tissues by qRT‒PCR. The results indicated that the mRNA expression levels of *MEG3* (Figure 1A) and *β*-Catenin (Figure 1B) were significantly lower, whereas NLRP3 (Figure 1C) expression was significantly higher in placental tissues of pregnant women with sPE than in normal pregnancies. Western blot analysis (Figures 1D,E) and immunofluorescence analyses (Figures 1F–H) further confirmed that the protein levels of *β*-Catenin and NLRP3 were the same as the changes in the mRNA levels. ELISA results revealed that the serum levels of IL-1β (Figure 1I) and TNF-α (Figure 1J) were significantly elevated in the sPE group versus normal pregnancies.

### H/R induces trophoblast dysfunction and inflammatory cytokine expression

**FIGURE 2:**
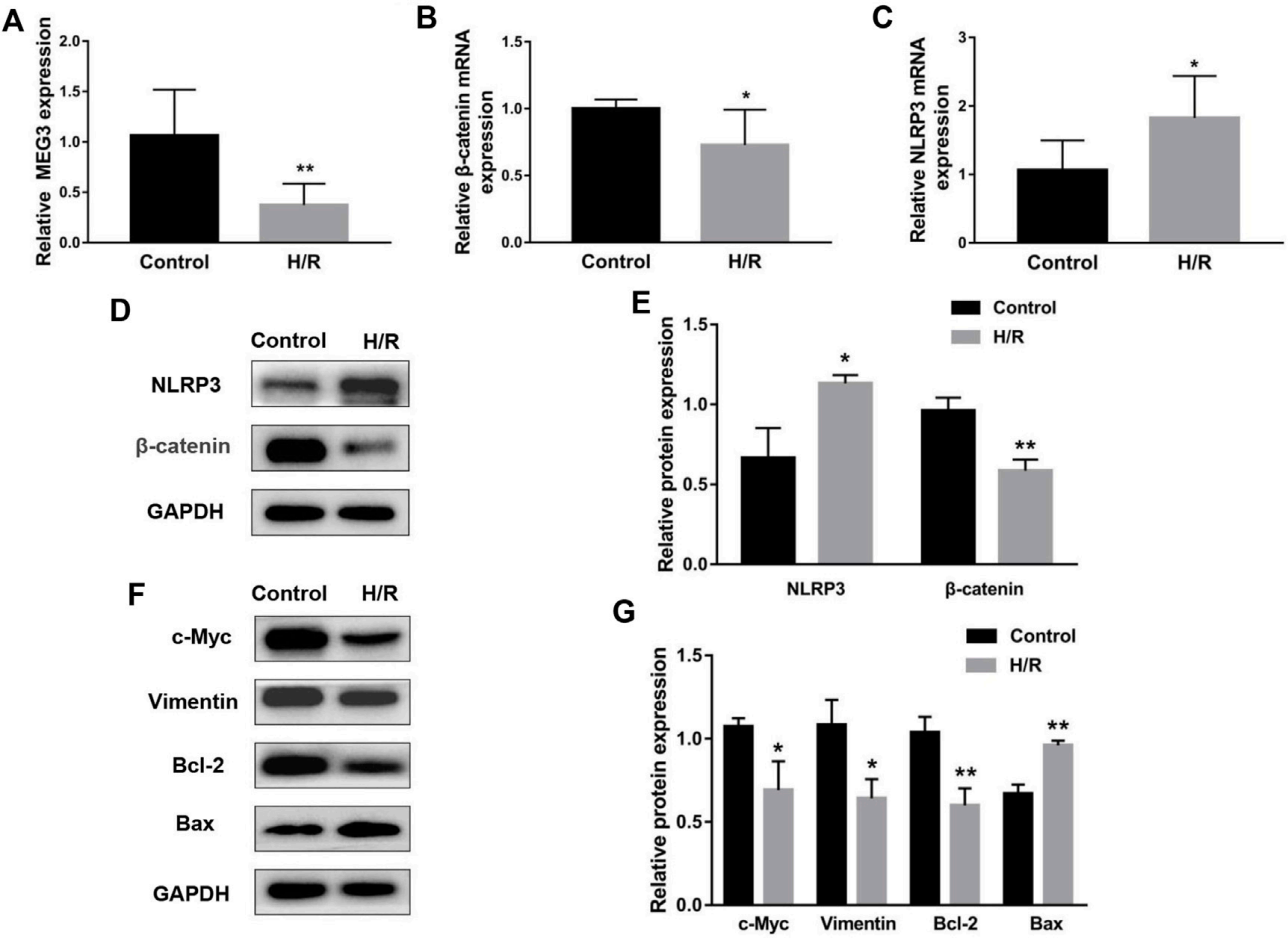
H/R treatment affected the expression of MEG3, *β*-Catenin and NLRP3 in trophoblast cells. H/R-induced changes in *MEG3* **(A)**, *β*-Catenin **(B)** and NLRP3 **(C)** mRNA levels were assessed by qRT‒PCR (*n* = 7/group). **(D,E)** H/R-induced changes in *β*-Catenin and NLRP3 protein levels were detected by Western blot (*n* = 3/group). **(F,G)** The expression levels of c-Myc, Vimentin, Bcl-2, and Bax induced by H/R were detected by Western blotting (*n* = 3/group). GAPDH was used as an endogenous control. *p < 0.05; **p < 0.01.

**FIGURE 3:**
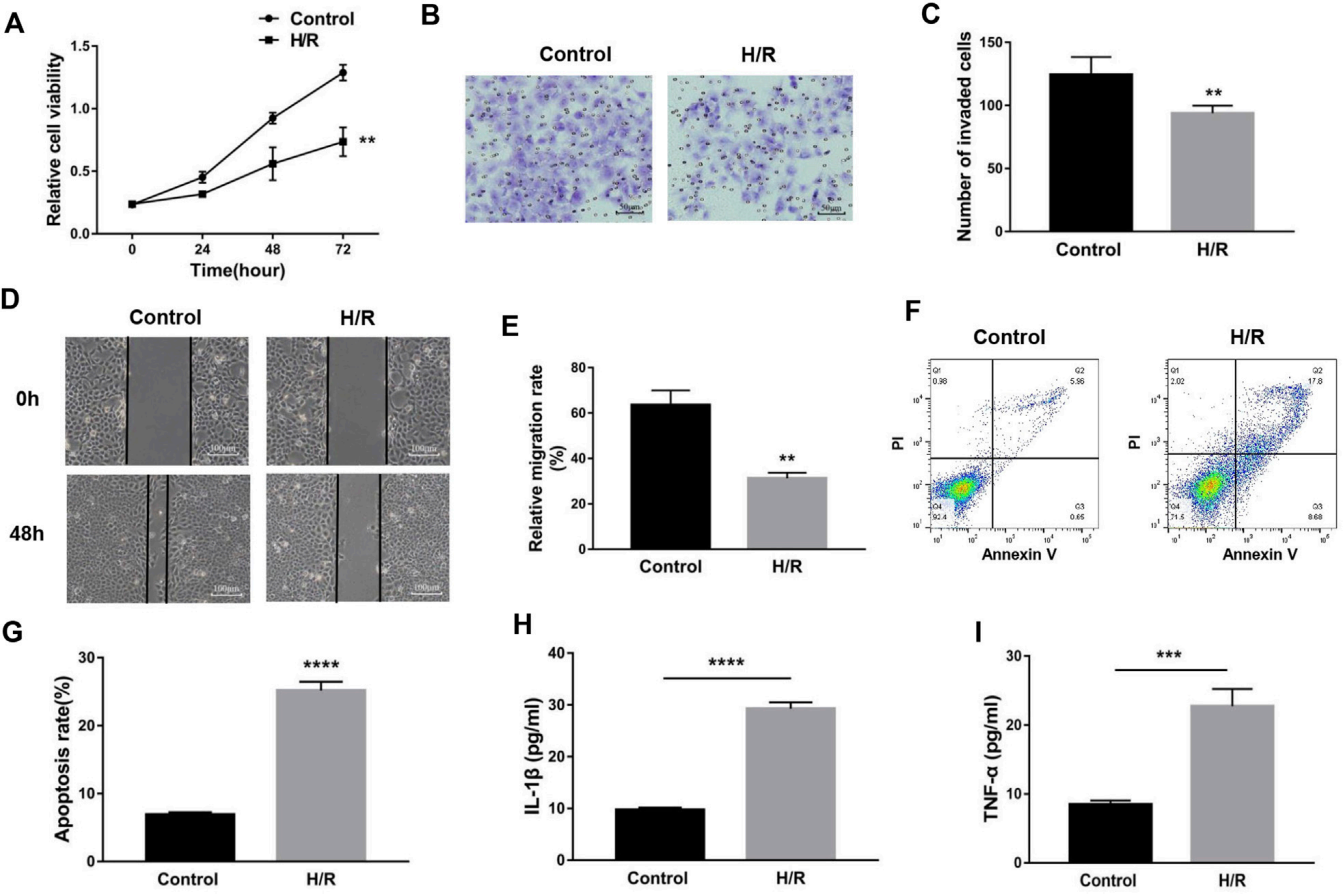
H/R-induced trophoblast dysfunction and inflammation were used to mimic preeclampsia. **(A)** Cell proliferation was determined by CCK-8 assay. **(B,C)** Cell invasion ability was determined by transwell assay. **(D,E)** Cell migration ability was determined by scratch assay. **(F,G)** The apoptosis rate was measured by flow cytometry. ELISA was used to detect IL-1β **(H)** and TNF-α **(I)** in the cell supernatant. *n* = 3/group. **p < 0.01; ***p < 0.001; ****p < 0.0001.

To simulate the microenvironment of PE *in vitro*, trophoblasts were cultured with H/R treatment. Compared to the control group, *MEG3* and *β*-Catenin levels were downregulated and NLRP3 expression was upregulated in HTR-8/SVneo cells cultured in H/R, as shown by qRT-PCR (Figures 2A–C) and Western blotting assays (Figures 2D,E). Subsequently, CCK-8 (Figure 3A), transwell (Figures 3B,C), wound healing (Figures 3D,E), and flow cytometry (Figures 3F,G) assays were conducted to assess proliferation, invasion, migration and apoptosis, and the results were consistent with the significantly decreased expression of the viability-related markers c-Myc and Vimentin and the antiapoptosis indicator Bcl-2, as well as the dramatically increased levels of Bax in the H/R group compared to the control group (Figures 2F,G). ELISA analysis of the supernatant of HTR8/SVneo medium revealed that H/R treatment significantly enhanced the levels of IL-1β (Figure 3H) and TNF-α (Figure 3I). Taken together, our results suggest that it is reasonable to use H/R to construct a model of preeclampsia.

### Upregulation of maternally expressed gene 3 relieves trophoblast dysfunction and inflammatory cytokine expression induced by H/R treatment

**FIGURE 4:**
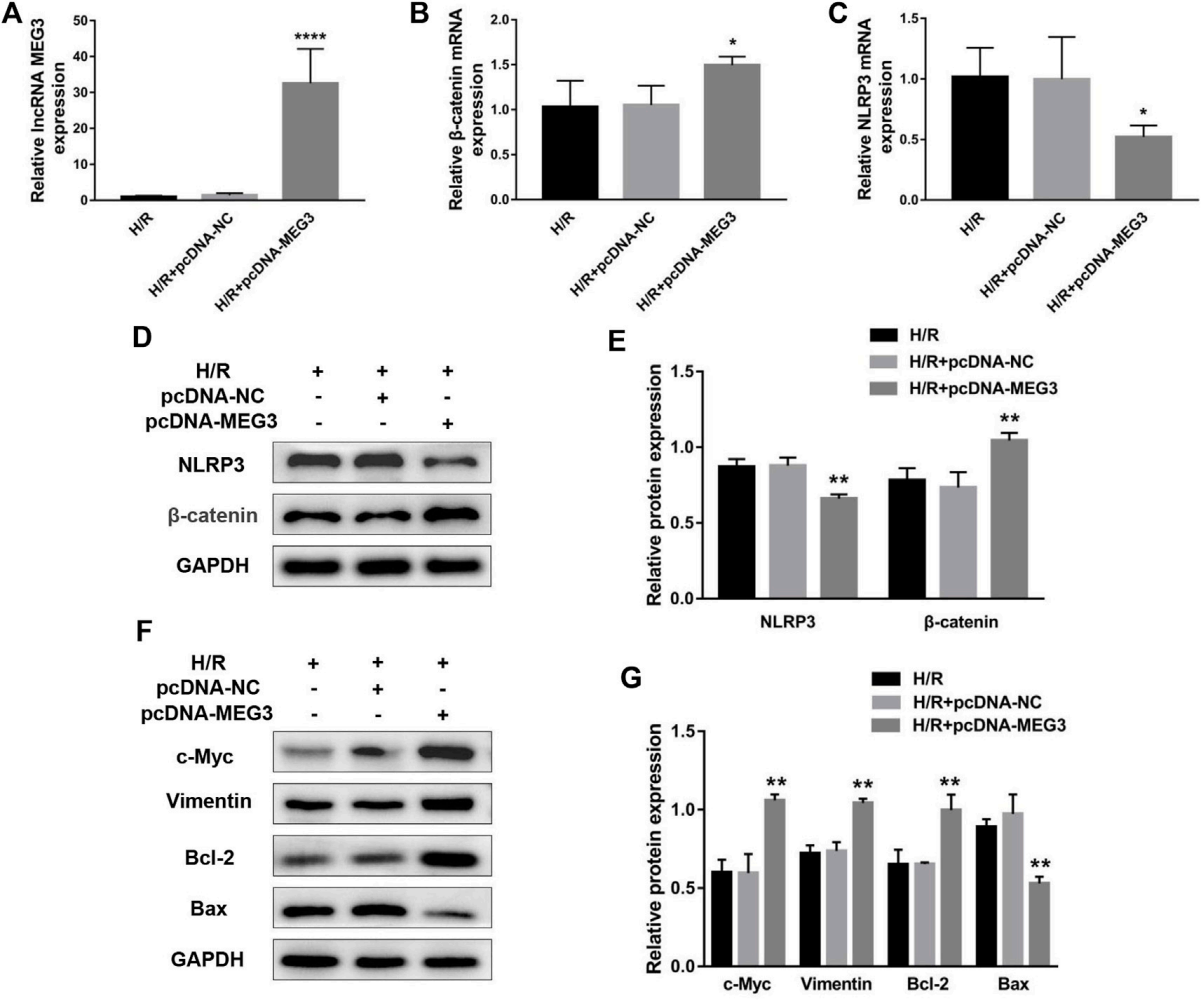
The overexpression of MEG3 altered the expression levels of *β*-Catenin and NLRP3 in trophoblast cells. The mRNA expression levels of *MEG3* **(A)**, *β*-Catenin **(B)** and NLRP3 **(C)** after overexpression of *MEG3* were assessed by qRT‒PCR (*n* = 4/group). **(D,E)** The protein expression levels of *β*-Catenin and NLRP3 after overexpression of *MEG3* were detected by Western blot (*n* = 3/group). **(F,G)** The expression levels of c-Myc, Vimentin, Bcl-2, and Bax were assessed by Western blotting (*n* = 3/group). GAPDH was used as an endogenous control. *p < 0.05; **p < 0.01; ****p < 0.0001.

**FIGURE 5:**
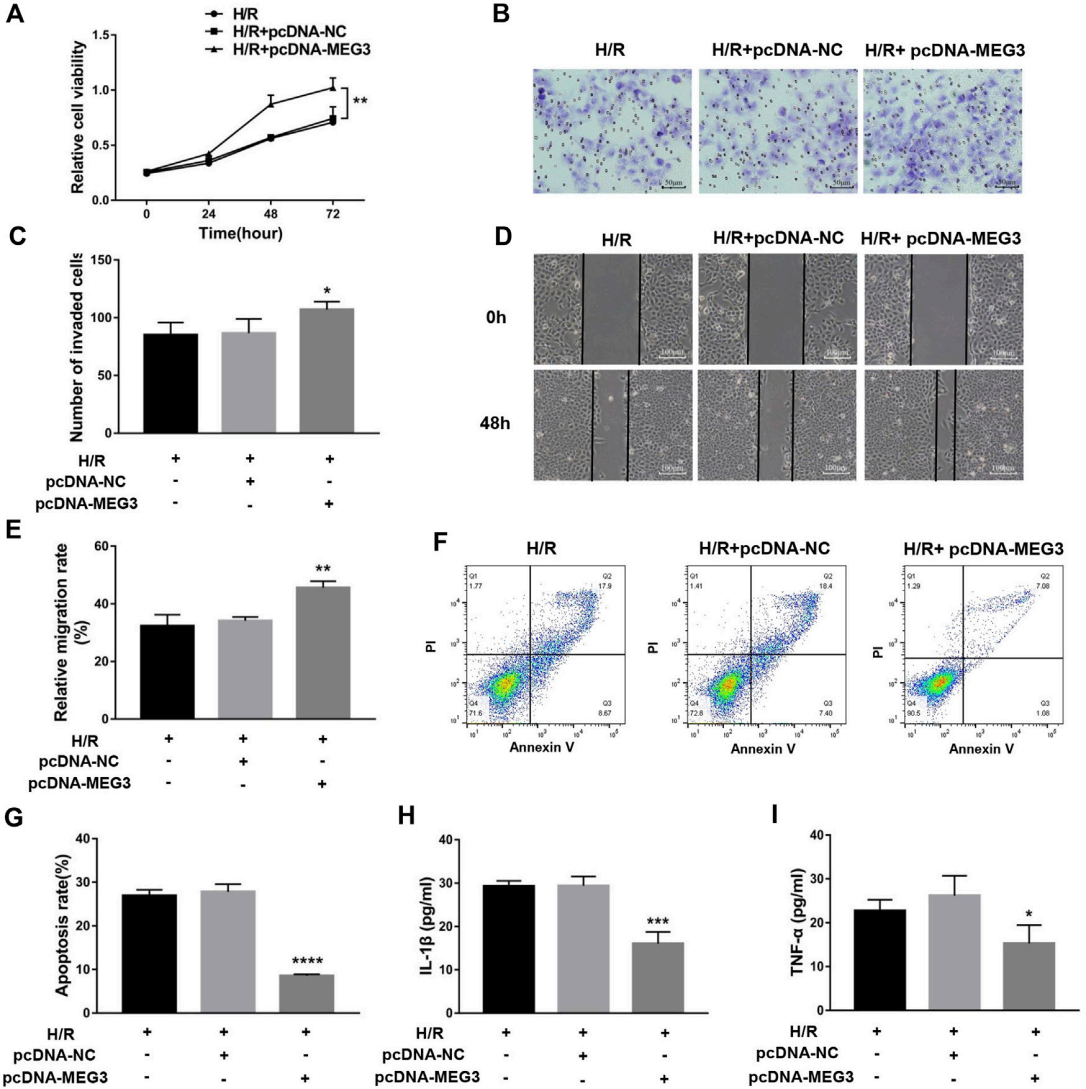
The overexpression of MEG3 overcame H/R-induced trophoblast dysfunction and inflammatory cytokine secretion. **(A)** Cell proliferation was determined by CCK-8 assay. **(B,C)** Cell invasion ability was determined by transwell assay. **(D,E)** Cell migration ability was determined by scratch assay. **(F,G)** The apoptosis rate was measured by flow cytometry. ELISA was used to measure IL-1β **(H)** and TNF-α **(I)** levels in the cell supernatant. *n* = 3/group. *p < 0.05; **p < 0.01; ***p < 0.001; ****p < 0.0001.

To uncover the specific effect of *MEG3* on trophoblast cell function, *MEG3* expression levels were upregulated by introducing pcDNA-*MEG3*. Compared to the H/R and H/R + NC groups, HTR8/SVneo cells transfected with pcDNA-*MEG3* after H/R manifested enhanced mRNA levels of *MEG3* (Figure 4A) and *β*-Catenin (Figure 4B) and significantly suppressed mRNA levels of NLRP3 (Figure 4C). At the protein level, the changes in *β*-Catenin and NLRP3 were consistent with the changes in their mRNA levels (Figures 4D,E). Furthermore, the results revealed that the proliferation (Figure 5A), invasion (Figures 5B,C) and migration (Figures 5D,E) of trophoblast cells were significantly promoted, whereas the apoptosis rate (Figures 5F,G) and inflammation (Figures 5H,I) were significantly reduced in the *MEG3* overexpression group versus the other two groups. Related proteins varied similarly to these results (Figures 4F,G).

### Maternally expressed gene 3 regulates trophoblast function through the Wnt/*β*-Catenin signaling pathway

**FIGURE 6:**
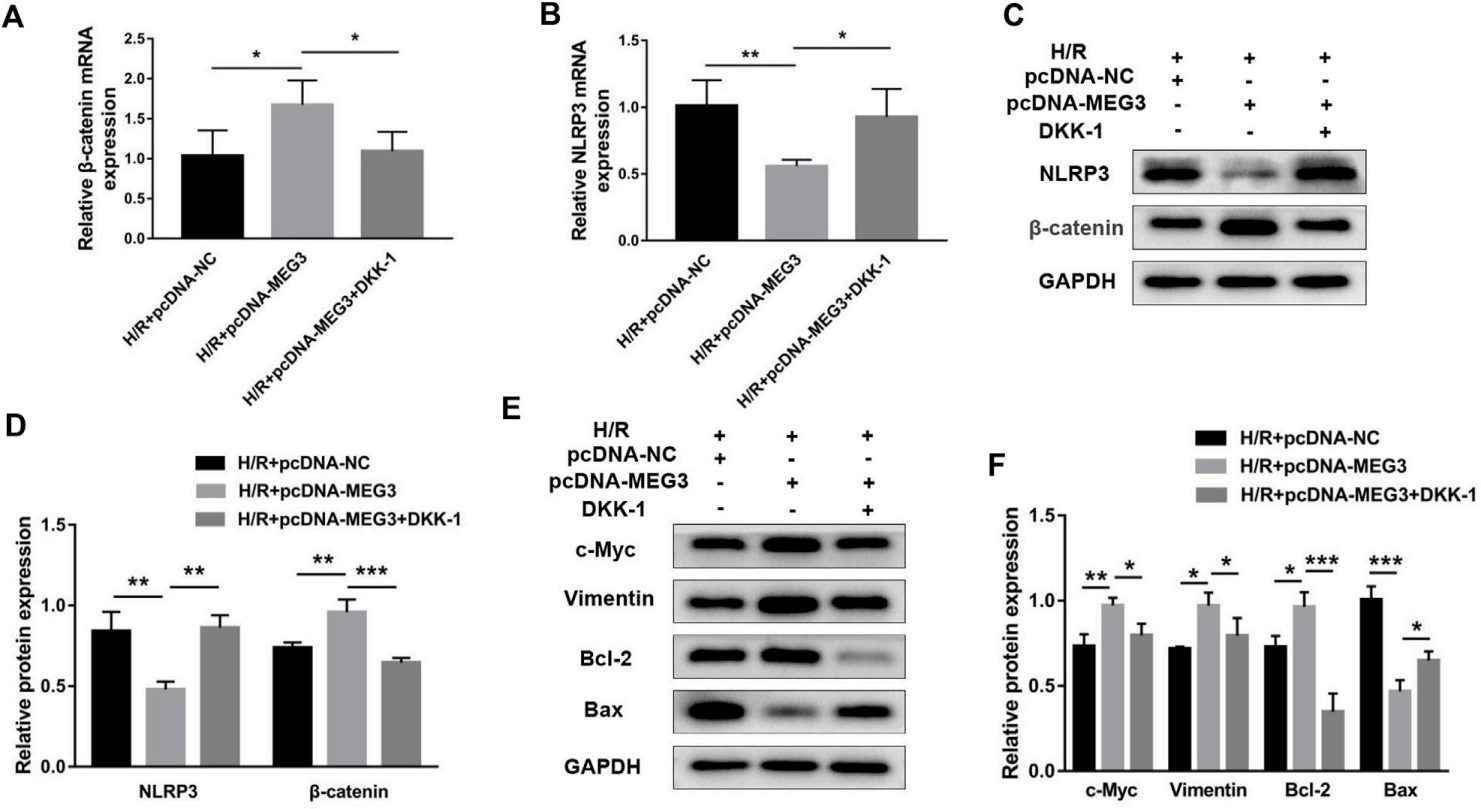
Blocking Wnt/*β*-Catenin signaling reversed the effect of MEG3 on the expression levels of *β*-Catenin and NLRP3. qRT‒PCR analysis of *β*-Catenin **(A)** and NLRP3 **(B)** in HTR8/SVneo cells overexpressing *MEG3* treated with DKK-1 (*n* = 4/group). **(C,D)** Western blot analysis of *β*-Catenin and NLRP3 in HTR8/SVneo cells overexpressing *MEG3* treated with DKK-1 (*n* = 3/group). **(E,F)** Western blot analysis of the expression levels of c-Myc, Vimentin, Bcl-2, and Bax (*n* = 3/group). GAPDH was used as an endogenous control. *p < 0.05; **p < 0.01; ***p < 0.001.

**FIGURE 7:**
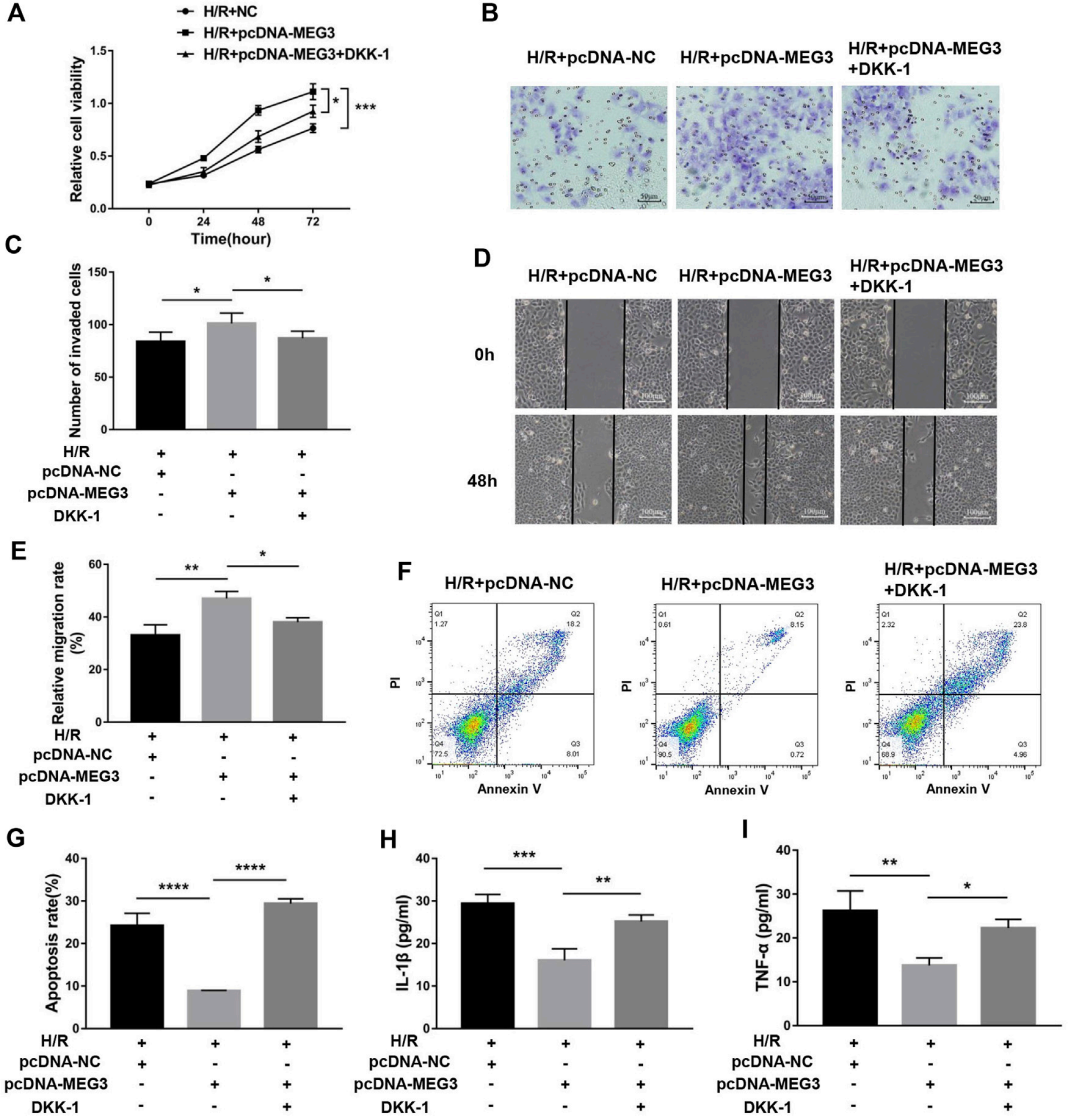
Blocking Wnt/*β*-Catenin signaling disrupted the effect of MEG3 on trophoblast function. **(A)** Cell proliferation was determined by CCK-8 assay. **(B,C)** Cell invasion ability was determined by transwell assay. **(D,E)** Cell migration ability was determined by scratch assay. **(F,G)** The apoptosis rate was measured by flow cytometry. ELISA was used to measure IL-1β **(H)** and TNF-α **(I)** levels in the cell supernatant. *n* = 3/group. *p < 0.05; **p < 0.01; ***p < 0.001; ****p < 0.0001.

To verify the contribution of the Wnt/*β*-Catenin pathway to trophoblast function and the *MEG3* regulatory pathway, we treated HTR8/SVneo cells with DKK-1, a Wnt/*β*-Catenin pathway inhibitor. We found that the *MEG3*-induced upregulation of *β*-Catenin and downregulation of NLRP3 were reversed by DKK-1 at the mRNA (Figures 6A,B) and protein levels (Figures 6C,D). Similar reverse effects by DKK-1 were also observed in the aspects of proliferation (Figure 7A), invasion (Figures 7B,C), migration (Figures 7D,E), apoptosis (Figures 7F,G) and related proteins (Figures 6E,F), as well as the inflammatory factors IL-1β (Figure 7H)and TNF-α (Figure 7I). These results indicate that *MEG3* contributes to improving trophoblast cell function through the Wnt/*β*-Catenin pathway.

### Nod-like receptor pyrin domain-containing 3 is part of the maternally expressed gene 3/Wnt/*β*-Catenin axis that regulates trophoblast function

**FIGURE 8:**
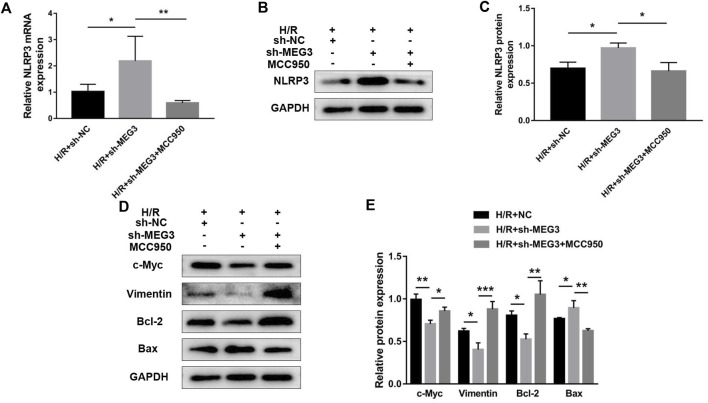
Suppressing NLRP3 expression reversed the effect of MEG3 knockdown on the expression level of NLRP3. qRT‒PCR [**(A)**; *n* = 4/group] and Western blotting [**(B,C)**; *n* = 3/group] were performed to evaluate NLRP3 mRNA and protein levels in *MEG3* knockout HTR8/SVneo cells treated with MCC950. **(D,E)** Western blotting was performed to evaluate the expression levels of c-Myc, Vimentin, Bcl-2, and Bax (*n* = 3/group). GAPDH was used as an endogenous control. *p < 0.05; **p < 0.01; ***p < 0.001.

**FIGURE 9:**
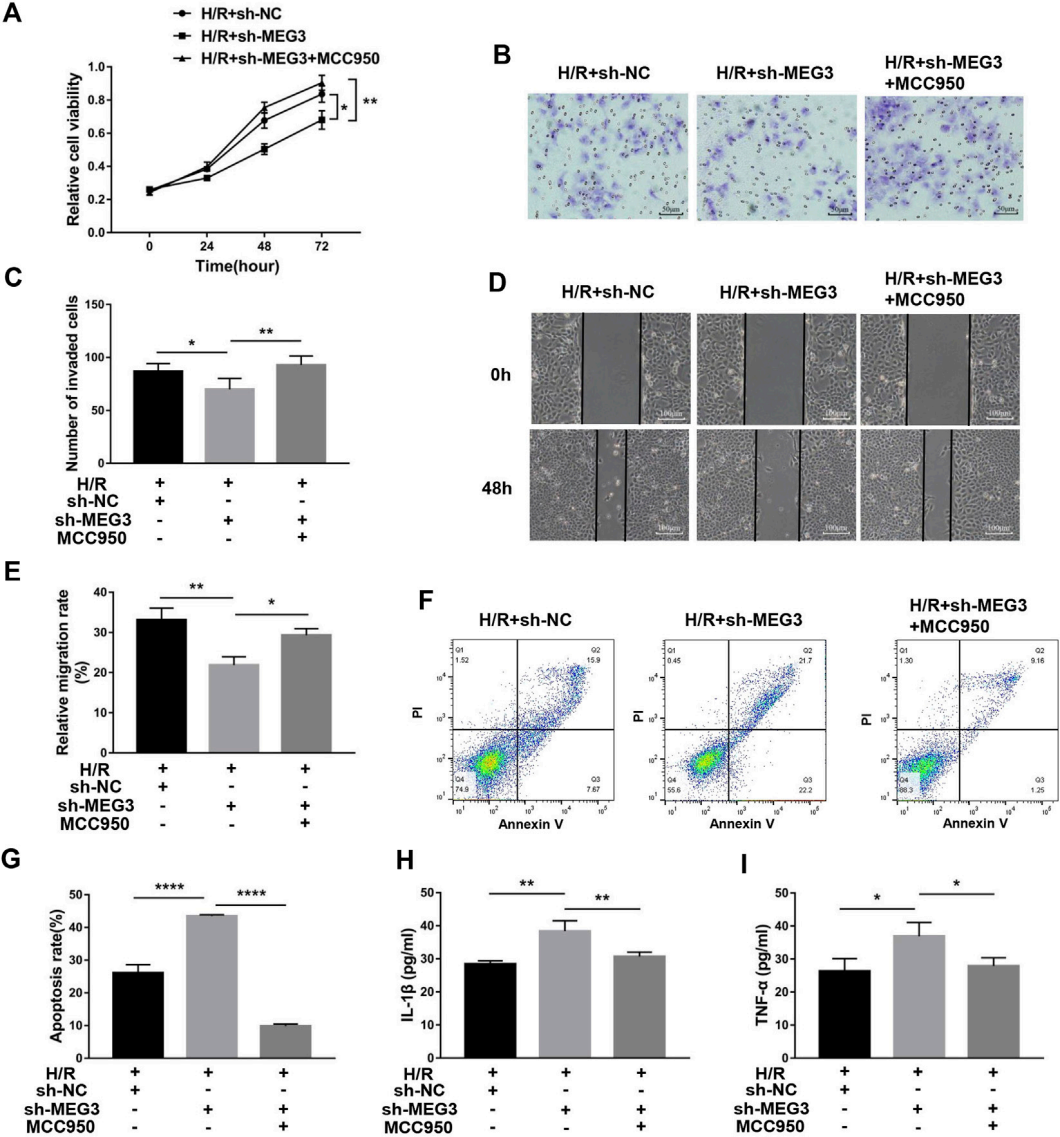
Suppressing NLRP3 expression alleviated the effect of *MEG3* knockdown on trophoblast function. **(A)** Cell proliferation was determined by CCK-8 assay. **(B,C)** Cell invasion ability was determined by transwell assay. **(D,E)** Cell migration ability was determined by scratch assay. **(F,G)** The apoptosis rate was measured by flow cytometry. ELISA was used to measure IL-1β **(H)** and TNF-α **(I)** levels in the cell supernatant. *n* = 3/group. *p < 0.05; **p < 0.01; ****p < 0.0001.

Next, we confirmed whether NLRP3 is involved in the effect of the *MEG3*/Wnt/*β*-Catenin axis on trophoblast function. Compared to the H/R group, targeting *MEG3* with shRNA substantially increased the expression of NLRP3 and further aggravated cell dysfunction induced by H/R. However, the upregulation of NLRP3 by sh-*MEG3* was reversed by the NLRP3 inhibitor MCC950 (Figures 8A–C). Moreover, HTR8/SVneo cells treated with MCC950 exhibited enhanced cell proliferation (Figure 9A), invasion (Figures 9B,C) and migration abilities (Figures 9D,E), evidently suppressing the apoptosis rate (Figures 9F,G) and inflammation level (Figures 9H,I), in addition to the same variation trend of related proteins (Figures 8D,E).

## Discussion

PE remains an important cause of maternal morbidity and mortality (Steegers et al., 2010). Although a great deal of research has been conducted, a cure eludes us due to limited understanding of its pathogenesis and pathophysiology (Skalis et al., 2019). At present, it is generally believed that the development of PE is related to the microenvironment of the maternal-fetal interface. Hypoxia and inflammation are two essential factors in the formation of the maternal-fetal interface microenvironment in preeclampsia. The underlying cause of hypoxia and inflammation is trophoblast dysfunction. However, the molecular mechanisms of trophoblast dysfunction in PE remain unclear.

In recent years, lncRNAs have been extensively studied for their relationship with trophoblast function and placental development. The study conducted earlier revealed that *MEG3* was underexpressed in preeclamptic placentas, which was in line with our results. Its downregulation has been demonstrated to decrease migration and invasion while promoting the apoptosis of trophoblasts (Zhang et al., 2015; Wang and Zou, 2020). Combined with our current research, these data illustrated that *MEG3* plays a vital role in the initiation and progression of PE through its effects on trophoblast cells. However, little is known about the specific downstream molecular mechanism of *MEG3* regulation in trophoblast cells. In the present investigation, we focused on the function and mechanism of lncRNA *MEG3* and indicated that *MEG3* reduces NLRP3 levels by targeting Wnt/*β*-Catenin and ultimately improves trophoblast cell function by promoting cell proliferation, migration, invasion and suppressing cell apoptosis and the inflammatory response.

First, the expression profiles of *β*-Catenin and NLRP3 in placental tissues were detected using PCR, Western blotting, and immunofluorescence. All of the experiments showed significantly lower expression of *β*-Catenin and higher expression of NLRP3 in preeclampsia placental tissues compared to normal pregnancies, implying that they might have functions related to preeclampsia. At the same time, the upregulation of the proinflammatory cytokines IL-1β and TNF-α in the serum of preeclampsia patients suggested the activation of systemic inflammation in preeclampsia patients. To investigate the detailed mechanisms, we performed *in vitro* experiments using HTR8/SVneo cell line.

H/R can give rise to oxidative stress, apoptosis and inflammation and is often used to mimic preeclampsia (Sagrillo-Fagundes et al., 2018). In our study, *MEG3* and *β*-Catenin expression decreased and NLRP3 expression increased in HTR-8/SVneo cells stimulated by H/R, similar to the levels observed in the placental tissue of preeclampsia. Subsequently, we verified that H/R induced trophoblast functional impairment and inflammation activation, which are not conducive to uterine spiral artery remodeling. Interestingly, pcDNA-*MEG3*-mediated overexpression of *MEG3* notably reversed changes in trophoblast cell function and molecular proteins triggered by H/R. These changes mainly manifest as elevated *β*-Catenin expression and decreased NLRP3 expression, intensifying the capacities of proliferation, migration and invasion as well as attenuating cell apoptosis and inflammation. *MEG3* overexpression also improves trophoblast cell function by regulating proteins related to proliferation, migration, invasion and apoptosis, such as c-Myc, Vimentin, Bcl-2, and Bax. Thus, we concluded that *MEG3* alleviates preeclampsia progression not only by improving trophoblast dysfunction but also by inhibiting inflammatory activation.

Wnt/*β*-Catenin is a pivotal regulator of placental formation by facilitating the migration and invasion of trophoblast cells, which is corroborated by previous studies (Li et al., 2020; Chen et al., 2021; Zhang et al., 2022). Recently, a growing body of documents reported that *β*-Catenin is a target of *MEG3* and is involved in a variety of human diseases downstream of *MEG3*, including Wilms’ tumor, glioma, and oral squamous cell carcinoma (Gong and Huang, 2017; Liu et al., 2017; Teng et al., 2020). Similarly, as seen in a previous study, we found that *β*-Catenin was prominently strengthened after *MEG3* overexpression. To further verify whether *MEG3* affects the biological functions of trophoblast cells through the Wnt/*β*-Catenin signaling pathway, we used DKK1 to block Wnt signaling. Our data illustrated that blocking *β*-Catenin partially eliminated the recovery effect of *MEG3*, suggesting that *β*-Catenin mediates the regulation of *MEG3* on trophoblast cell function.

Consistent with previous reports, we found that NLRP3 was substantially increased in PE and was relevant to impaired trophoblast function and aggravated inflammation (Li et al., 2021; Weel et al., 2017; Liu et al., 2019). Identified that NLRP3, which is downstream of the Wnt/*β*-Catenin pathway, is involved in the regulation of melatonin on osteoblast differentiation Xu et al. (2018). As per these reports, we hypothesized that the regulation of trophoblast cells by *MEG3*/Wnt/*β*-Catenin may be mediated by NLRP3. Thus, we used an NLRP3-specific inhibitor, MCC950, to downregulate NLRP3 levels and observed that when NLRP3 expression was decreased, superimposed injury of trophoblast cells by sh-*MEG3* and H/R was greatly weakened.

The present study has several limitations. First, the gestational age of preeclampsia was earlier than that of the normal pregnancy group due to the difference in optimal termination time. Second, further confirmation of this discovery is needed in animal models of preeclampsia and other trophoblast cell lines. Third, the correlation between placental tissue apoptosis and *MEG3* expression should be investigated to validate our results in the HTR8/SVneo cell line. Additionally, the data generated by our experiments only illustrated the critical role of *MEG3* in the placenta of PE. Maternal vascular wall dysfunction caused by placental factors is also considered to be the key pathogenesis of PE (Tomimatsu et al., 2019). Thus, the possible downstream pathways and regulatory mechanisms of *MEG3* involved in maternal and placental vascular dysfunction in PE deserve to be further explored in future work.

Collectively, our research implied low levels of *MEG3* and *β*-Catenin and high levels of NLRP3 in the placental tissue of PE and demonstrated that *MEG3* can participate in the proliferation, migration, invasion, apoptosis and inflammatory response of trophoblast cells through the Wnt/*β*-Catenin/NLRP3 pathway. Our research revealed a promising target for the clinical treatment of preeclampsia and a new direction for the study of its pathogenesis.

## Data Availability

The raw data supporting the conclusion of this study are available from the corresponding author.
